# Optic Nerve Sheath Diameter and Transcranial Doppler Pulsatility Index for Non-Invasive ICP Assessment in Acute Intracerebral Hemorrhage

**DOI:** 10.3390/brainsci16060553

**Published:** 2026-05-22

**Authors:** Nguyen Van Tuyen, Nguyen Hoang Ngoc, Nguyen Thị Cuc, Nghiem Xuan Hoan

**Affiliations:** 1 Department of Stroke, Institute of Neurology, 108 Military Central Hospital, Hanoi 100000, Vietnam; bstuyena21@gmail.com (N.V.T.); hoangngocdqn108@gmail.com (N.H.N.); cucnguyenqy41@gmail.com (N.T.C.); 2 University of Medicine and Pharmacy, Vietnam National University, Hanoi 100000, Vietnam; 3 Vietnamese-German Center for Medical Research (VG-CARE), 108 Military Central Hospital, Hanoi 100000, Vietnam; 4 Department of Science and Technology Management, 108 Military Central Hospital, Hanoi 100000, Vietnam

**Keywords:** intracranial pressure, optic nerve sheath diameter, transcranial Doppler, intracerebral hemorrhage, non-invasive monitoring, stroke

## Abstract

**Highlights:**

**What are the main findings?**

- ONSD and TCD-derived PI enable non-invasive assessment of elevated ICP in intracerebral hemorrhage.
- Integration of ONSD, PI, and clinical variables in machine learning enhanced both ICP detection and outcome prediction.

**What are the implications of the main findings?**

- Combining ONSD, PI, and clinical factors may improve early mortality prediction and risk stratification of ICH.
- This non-invasive assessment may complement clinical evaluation where invasive ICP monitoring is limited.

**Abstract:**

**Background:** Intracranial hypertension is a critical complication of acute intracerebral hemorrhage (ICH), contributing to high early mortality and poor functional outcomes. Invasive intracranial pressure (ICP) monitoring remains the gold standard but carries procedural risks and is resource-intensive. This study evaluated the diagnostic and prognostic utility of optic nerve sheath diameter (ONSD) ultrasonography and transcranial Doppler (TCD)-derived pulsatility index (PI) as non-invasive ICP surrogates in patients with severe ICH. **Methods:** A prospective observational study was conducted in 42 patients with acute ICH who underwent concurrent invasive ICP monitoring and serial ONSD/PI measurements at 10 time points (T0–T9) between October 2021 and August 2024. Diagnostic performance was assessed using measurement-level receiver operating characteristic (ROC) curve analysis. Exploratory early mortality prediction was evaluated using random forest machine learning models incorporating ONSD, PI, age, and sex. **Results:** A total of 274 paired ONSD–PI–ICP measurements were obtained. Both ONSD and PI showed moderate positive correlations with invasive ICP (rho = 0.49 and 0.43, respectively; *p* < 0.001). ONSD demonstrated superior diagnostic accuracy for detecting ICP ≥ 20 mmHg (AUC = 0.83; optimal threshold: 5.88 mm; sensitivity: 81%; specificity: 82%) compared to PI (AUC = 0.75). In exploratory random forest analyses, the combined ONSD–PI model showed high apparent discrimination for elevated ICP detection (AUC = 0.98), while the model incorporating ONSD, PI, age, and sex showed promising but potentially optimistic discrimination for early mortality prediction (AUC = 0.95). These machine learning results should be interpreted cautiously because of the small sample size, repeated-measurement structure, measurement-level data partitioning, and limited number of early deaths. **Conclusions:** ONSD ultrasonography and TCD-derived PI showed promising performance as non-invasive ICP markers in severe acute ICH. However, because of the small sample size, repeated-measurement design, measurement-level analyses, and exploratory nature of the machine learning models, these findings require validation in larger external cohorts before routine clinical implementation.

## 1. Introduction

Intracranial hypertension is a critical and life-threatening complication commonly observed in patients with acute intracerebral hemorrhage (ICH), significantly contributing to high rates of early mortality and severe disability [1]. The global burden of ICH is disproportionately high in low- and middle-income countries, where the prevalence of vascular risk factors, such as hypertension, is elevated [2,3]. The pathophysiological processes underlying ICH involve mass effects from expanding hematomas, secondary cerebral edema, and obstruction of cerebrospinal fluid pathways, all of which exacerbate the elevation of intracranial pressure (ICP) [1,4]. Early and accurate detection of increased ICP is crucial for guiding therapeutic interventions and improving patient outcomes.

Invasive monitoring techniques, particularly intraventricular catheter placement, are considered the gold standard for ICP measurement [1,4,5,6]. These methods allow for both precise monitoring and therapeutic CSF drainage. However, invasive approaches carry inherent risks, including infection, hemorrhage, and procedural complications, which may offset their benefits in certain patient populations. Furthermore, these methods are cost-intensive and often unavailable in resource-constrained settings [1,4]. As such, there is a growing need for non-invasive, reliable, and accessible alternatives for ICP monitoring, particularly in contexts where invasive techniques are impractical or contraindicated [3].

Among non-invasive techniques, optic nerve sheath diameter (ONSD) ultrasonography and transcranial Doppler (TCD) sonography have emerged as promising modalities for assessing elevated ICP [6,7]. ONSD ultrasonography leverages the anatomical relationship between the optic nerve sheath and ICP, wherein increased ICP leads to distension of the sheath due to transmission of pressure through the subarachnoid space [2,8]. On the other hand, TCD sonography measures cerebral blood flow dynamics, providing indirect information about cerebrovascular resistance and pulsatility index (PI), both of which correlate with ICP levels [3,8]. These methods are advantageous due to their non-invasive nature, repeatability, low cost, and portability, making them particularly suitable for bedside applications in critically ill patients [2].

Preliminary studies have highlighted the utility of ONSD and TCD in predicting ICP in various neurological conditions, mostly reported in traumatic brain injury. For instance, a systematic review and meta-analysis of 222 patients with TBI revealed that ultrasonography performed well in detecting an increased ICP, with an overall pooled sensitivity of 0.82 (95% CI: 0.75–0.88) and a specificity of 0.82 (95% CI: 0.71–0.90) [5]. In addition, TCD-derived PI has shown promise as an indicator of ICP, with studies demonstrating significant associations between elevated PI and poor clinical outcomes in patients with ICH [1,8]. Despite their potential, the diagnostic performance of ONSD and TCD in predicting intracranial hypertension remains variable across studies. Research has demonstrated moderate to strong correlations between these non-invasive indices and invasive ICP measurements, yet questions about sensitivity, specificity, and optimal cutoff values persist [9].

Combining these two modalities has the potential to improve diagnostic accuracy by addressing the limitations of each method when used independently [3]. However, evidence supporting the combined use of these techniques remains limited, particularly in the context of hemorrhagic stroke, which poses unique challenges due to its acute and dynamic course [3]. In Vietnam, ICH represents a significant public health challenge, with high mortality and morbidity rates attributed to delayed diagnosis and suboptimal management of intracranial hypertension. Current guidelines emphasize the importance of ICP monitoring in guiding treatment decisions for patients with severe brain injuries, including ICH. However, invasive ICP monitoring is often constrained by limited resources, technical expertise, and risks associated with its use [1,4]. Non-invasive techniques such as ONSD and TCD, if validated, could provide an effective solution to these challenges, enabling timely and accurate detection of intracranial hypertension without the need for invasive procedures [2,3].

Although ONSD ultrasonography and TCD-derived PI have been investigated in several neurocritical care populations, comprehensive evidence integrating these two bedside ultrasound modalities specifically in Vietnamese patients with acute ICH remains limited. This study therefore sought to evaluate the feasibility and potential diagnostic value of a non-invasive bedside approach for assessing intracranial hypertension in Vietnamese patients with severe acute ICH. The specific objectives of this study were (i) to evaluate the sensitivity and specificity of combined ONSD and TCD measurements compared to invasive ICP monitoring; (ii) to analyze the correlation between ONSD, TCD-derived PI (PI), and invasive ICP levels; (iii) to determine optimal threshold values for ONSD and PI in predicting increased intracranial pressure.

## 2. Materials and Methods

### 2.1. Study Design and Setting

This prospective, longitudinal observational study was conducted at the Department of Stroke, Institute of Neurology, 108 Military Central Hospital, Hanoi, Vietnam, between October 2021 and August 2024. The study adhered to the Standards for Reporting of Diagnostic Accuracy Studies (STARD 2015) guidelines for the diagnostic accuracy component and to the Strengthening the Reporting of Observational Studies in Epidemiology (STROBE) checklist for the prospective cohort design.

### 2.2. Study Participants

#### 2.2.1. Eligibility Criteria

Eligible patients were adults aged ≥18 years admitted with confirmed acute intracerebral hemorrhage (ICH) within 10 days of symptom onset and a clinical indication for invasive intracranial pressure (ICP) monitoring. Inclusion required a Glasgow Coma Scale (GCS) score ≤ 8, or a GCS score of 9–12 accompanied by at least one severe radiological finding on computed tomography (CT): intraventricular hemorrhage (IVH) with a Graeb score ≥ 8, parenchymal hematoma volume ≥ 30 mL, or subarachnoid hemorrhage (SAH) classified as Fisher grade 4. All eligible patients were required to complete both TCD and ONSD ultrasound assessments.

Exclusion criteria comprised: (i) absence of adequate temporal bone acoustic windows precluding TCD insonation; (ii) contraindications to ONSD ultrasonography (ocular trauma, optic neuritis, congenital ocular anomalies); (iii) contraindications to invasive ICP monitoring including local infection, coagulopathy, or severe anemia (hemoglobin < 80 g/L or hematocrit ≤ 27%); (iv) extensive scalp defects; (v) uncorrected hemodynamic instability (systolic blood pressure < 90 mmHg); (vi) confounding intracranial pathologies such as primary brain tumors or chronic hydrocephalus; and (vii) refusal to provide informed consent.

#### 2.2.2. Enrollment

**Figure 1:**
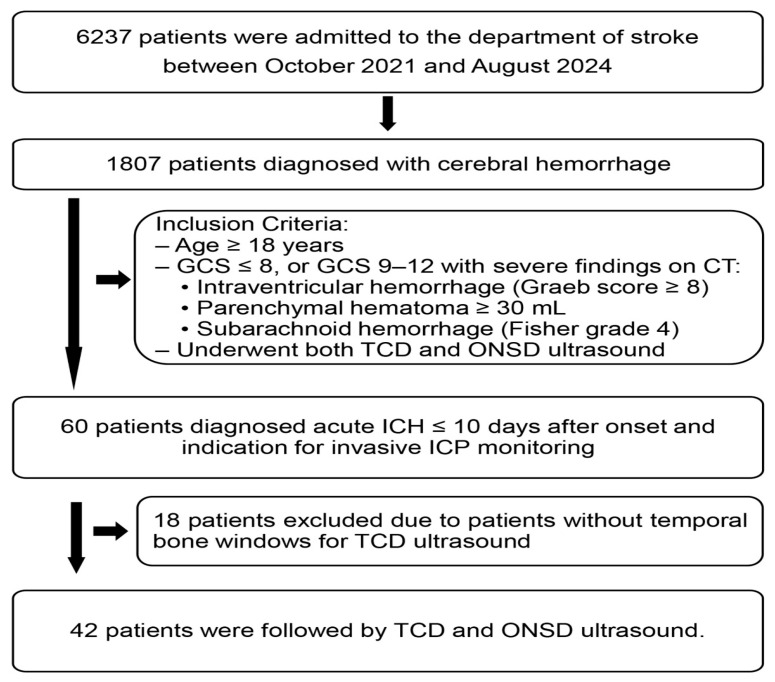
Flowchart of patient selection.

Of 6237 patients admitted during the study period, 1807 were diagnosed with cerebral hemorrhage. Sixty patients fulfilled eligibility criteria for acute ICH with an indication for invasive ICP monitoring. After excluding 18 patients who lacked adequate temporal bone windows for TCD assessment, 42 patients were included in the final analysis (Figure 1).

### 2.3. Invasive ICP Monitoring

Catheter placement was performed by attending neurosurgical specialists. Intraparenchymal or intraventricular ICP monitoring was conducted using the Camino^®^ fibre-optic pressure sensor system (Natus Medical Inc., Pleasanton, CA, USA) or the Integra Neurosciences pressure transducer system (Integra LifeSciences Corp., Plainsboro, NJ, USA). Patient management conformed to the 2015 AHA/ASA stroke management guidelines. In accordance with Brain Trauma Foundation recommendations, an ICP threshold of ≥20 mmHg was adopted as the criterion for clinically significant intracranial hypertension.

ICP was recorded at T0 (baseline, immediately prior to catheter placement) and at T1 through T9 (once each morning on consecutive post-catheter days 2–10). Data collection for a given patient was discontinued upon death, hospital discharge, or catheter removal. Catheter removal was indicated when ICP had been within normal limits for ≥2 consecutive days, when catheter-site infection was detected, or when the catheter had been in situ for ≥14 days.

### 2.4. ONSD and TCD-PI Measurement Procedures

All ultrasound measurements were performed by trained clinicians using a portable Digital-Life ultrasound system, according to a pre-specified written protocol to minimize inter-observer variability.

ONSD: Patients were positioned supine at 30° head-of-bed elevation. ONSD was measured in B-mode with an 8.5 MHz linear transducer applied to the closed upper eyelid with acoustic gel to avoid direct ocular pressure. The measurement site was located 3 mm posterior to the posterior globe surface, perpendicular to the optic nerve long axis, using the outer-to-outer hyperechoic border of the nerve sheath as the anatomical landmark. Three independent measurements were obtained per eye; the highest value per eye was retained. The mean of both eyes at each time point constituted the final ONSD value for analysis.

PI: TCD of the middle cerebral artery (MCA) was performed immediately following ONSD assessment using a 2 MHz phased-array transducer via the transtemporal window at an insonation depth of 45–65 mm. The pulsatility index was computed as $PI=\frac{PSV-EDV}{MFV}$, where PSV = peak systolic velocity, EDV = end-diastolic velocity, and MFV = mean flow velocity. Three measurements were obtained bilaterally; the highest value per side was retained. The mean of both MCA measurements at each time point constituted the final PI value.

ONSD and PI measurements were performed concurrently with each invasive ICP recording at all 10 time points (T0–T9), yielding 274 paired ONSD–PI–ICP triplets across the cohort. Serial ONSD and TCD-derived PI assessments were performed concurrently with invasive ICP recordings at predefined time points, allowing repeated bedside evaluation of the relationship between non-invasive ultrasound markers and invasive ICP during the acute monitoring period.

### 2.5. Clinical, Radiological, and Outcome Variables

Baseline data recorded at admission included: demographic variables (age, sex), neurological severity scores (GCS, NIH Stroke Scale [NIHSS]), comorbidities (hypertension, diabetes mellitus, prior stroke, renal failure, hepatic cirrhosis), hemorrhage characteristics on CT (type, hematoma volume, Graeb score for IVH, Fisher grade for SAH), vascular anatomy by CT angiography or digital subtraction angiography, and surgical interventions performed (external ventricular drainage [EVD], hematoma drainage [HD], or combined). The type of ICP monitoring catheter (intraventricular vs. intraparenchymal) was recorded for all patients.

Primary outcome measures were: (i) early mortality within 10 days of symptom onset and (ii) mortality within 30 days of symptom onset. Functional outcome was assessed by the modified Rankin Scale (mRS) at 30 days.

### 2.6. Statistical Analysis

All statistical analyses were performed using R software, version 4.3.2 (R Foundation for Statistical Computing, Vienna, Austria), an open-source programming language and statistical computing environment widely used for data analysis, visualization, and reproducible research [10]. Normality of continuous variables was assessed using the Shapiro–Wilk test [11]. Normally distributed data are presented as mean ± standard deviation (SD), non-normally distributed data as median and interquartile range (IQR), and categorical variables as frequencies and percentages.

Comparisons between groups were performed according to variable type, distribution, and sample size. For continuous variables that were not normally distributed or when the sample size was small, the Wilcoxon rank-sum test was used to compare two independent groups [12,13]. For categorical variables, the Chi-square test was used when the number of observations in each comparison category was sufficient, whereas Fisher’s exact test was applied when the number of observations in one or more categories was small [14,15].

Associations between ONSD, PI, and invasive ICP were quantified using Spearman’s rank correlation coefficient (rho) with 95% bootstrap confidence intervals. For all paired analyses, ONSD and PI values represented the mean of bilateral measurements at each time point. Because serial measurements were obtained from the same patients, these correlation analyses were interpreted as exploratory measurement-level analyses and *p*-values were interpreted cautiously. Repeated observations within the same patient may introduce within-subject correlation and affect the precision of correlation estimates and statistical significance. In future larger cohorts, mixed-effects models, generalized estimating equations, repeated-measures correlation, or other clustered analytical approaches should be used to account for intra-subject dependence.

Diagnostic accuracy of ONSD and PI for detecting ICP ≥ 20 mmHg was evaluated using measurement-level receiver operating characteristic (ROC) curve analysis. Discrimination was expressed as the area under the curve (AUC) with 95% confidence intervals. Optimal cut-off values were determined by maximizing the Youden Index (J = sensitivity + specificity − 1), with sensitivity, specificity, positive predictive value (PPV), and negative predictive value (NPV) reported at each threshold. Because repeated observations from the same patient are correlated, measurement-level ROC analysis may overestimate diagnostic performance compared with patient-level validation.

Machine learning was used as an exploratory approach to assess whether non-invasive ultrasound markers and simple clinical variables could improve classification of elevated ICP and early mortality. Random forest classifiers were developed for two outcomes: elevated ICP, defined as ICP ≥ 20 mmHg, and early mortality. Candidate predictors included ONSD, TCD-derived PI, age, and sex, selected based on clinical plausibility, preliminary univariate findings, and model parsimony. To reduce overfitting risk, the final models were restricted to a small number of clinically interpretable predictors. The dataset was divided into training and testing subsets using stratified sampling. Model performance was assessed using AUC, accuracy, sensitivity, and specificity, and variable importance was evaluated using mean decrease in accuracy and mean decrease in Gini impurity. Given the limited sample size, repeated-measurement structure, and small number of early deaths, these findings were interpreted as exploratory rather than confirmatory.

### 2.7. Ethics Statement

This study was conducted in accordance with the Declaration of Helsinki (revised 2013) and was approved by the Institutional Review Board of 108 Military Central Hospital, Hanoi, Vietnam (Approval No. 5582/GCN-BV, dated 29 November 2021). Written informed consent was obtained from all participants prior to enrollment. For patients who were incapacitated due to impaired consciousness or coma, written informed consent was obtained from their legally authorized representatives (spouse, parents, or adult children).

## 3. Results

### 3.1. Patient Enrollment and Demographic Characteristics

Between October 2021 and August 2024, a total of 6237 patients were admitted to the Department of Stroke. Of these, 1807 were diagnosed with cerebral hemorrhage, and 60 met the eligibility criteria for acute ICH with an indication for invasive ICP monitoring. Following the exclusion of 18 patients who lacked adequate temporal bone acoustic windows for TCD assessment, 42 patients were enrolled in the final analysis (Figure 1).

The cohort comprised predominantly middle-aged to elderly males (mean age: 57.98 ± 10.48 years; males: 85.7%). Hypertension was the most common comorbidity, present in 85.7% of patients, followed by diabetes mellitus (26.2%). The hemorrhage pattern at admission was characterized by a high frequency of intraventricular hemorrhage (IVH; 95.2%), with 64.3% classified as parenchymal hemorrhage and 33.3% as subarachnoid hemorrhage (all Fisher grade 4). Severe IVH (Graeb score ≥ 8) was identified in 40.5% of patients. Most patients presented with severe neurological impairment at admission: 71.4% had GCS scores of 3–8, and 66.7% had NIHSS scores of 21–42. External ventricular drainage (EVD) was performed in 85.7% of patients, with ventricular ICP monitoring in 88.1%. The 10-day and 30-day early mortality rates were 21.4% (9/42) and 35.7% (15/42), respectively. Among survivors at 30 days, 59.5% had significant functional disability (mRS 4–5), and only 4.8% achieved minimal disability (mRS 1–3). Full demographic and clinical characteristics are presented in Table 1.

### 3.2. ONSD Distribution and Its Correlation with Clinical and Radiological Severity

A total of 274 paired ONSD–PI–ICP measurements were collected across 10 planned time points (T0–T9) from 42 patients. The overall mean ONSD was 5.70 mm (range: 4.80–6.80 mm), which is notably higher than reference values reported in healthy populations, consistent with the presence of intracranial pathology in all enrolled subjects.

**Figure 2:**
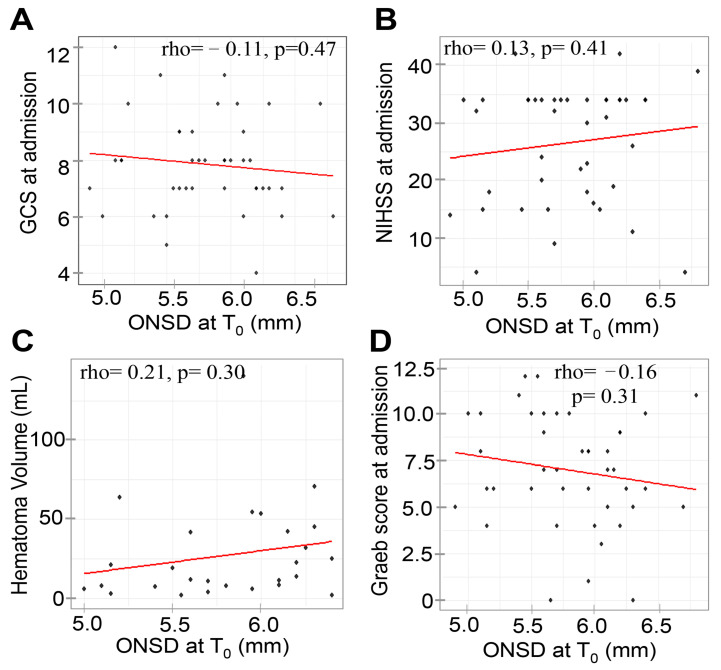
Correlation between ONSD and GCS, NIHSS, Graeb score at admission in patients with intracerebral hemorrhage. Scatter plots showed the correlation between ONSD and GCS (**A**), NIHSS (**B**), Hematoma volume (**C**), and Graeb score (**D**) in intracerebral hemorrhage at admission. Spearman’s correlation coefficient (rho) and corresponding *p*-values are displayed in each plot.

The correlations between ONSD at admission (T_0_) and clinical severity scores—GCS, NIHSS, hematoma volume, and Graeb score—are shown in Figure 2. Across all four comparisons, Spearman’s correlation coefficients were weak (|rho| < 0.30) and none reached statistical significance (GCS: rho = −0.11, *p* = 0.47; NIHSS: rho = 0.13, *p* = 0.41; hematoma volume: rho = 0.21, *p* = 0.30; Graeb score: rho = −0.16, *p* = 0.31). These findings indicate that single-point ONSD at admission does not reliably reflect initial neurological or hemorrhagic severity as assessed by conventional scales.

**Figure 3:**
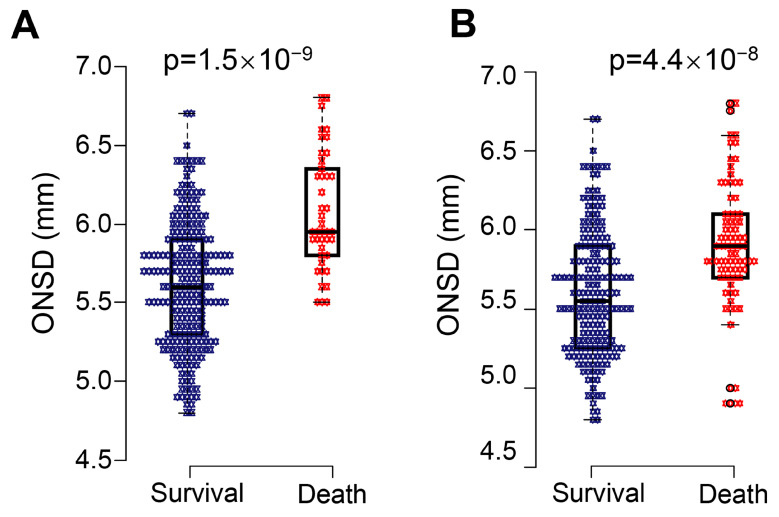
Distribution of ONSD measurements in patients with early mortality following intracerebral hemorrhage. Panel (**A**) represented 9 patients who died within 10 days post-ICH onset (or from admission). Panel (**B**) represented 15 patients who died within 1-month post-ICH onset (or from admission). The dot-box plots illustrated the distribution of ONSD values, with the embedded boxplots highlighting the median, interquartile range (IQR), and outliers.

The distribution of ONSD measurements according to early mortality outcome is presented in Figure 3. Patients who died within 10 days post-onset (panel A, *p* = 1.5 × 10^−9^) and within 30 days (panel B, *p* = 4.4 × 10^−8^) exhibited significantly higher ONSD values compared to survivors, based on the Wilcoxon rank-sum test. Although distributional overlap was observed between groups, these results indicate a statistically robust association between elevated ONSD and early mortality.

### 3.3. Diagnostic Performance of ONSD for Detecting Elevated Intracranial Pressure

**Figure 4:**
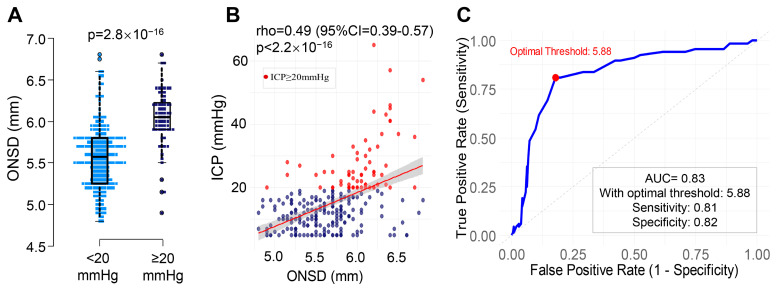
Diagnostic value of ONSD in diagnosing elevated ICP. Panel (**A**) displayed ONSD measurements between two ICP groups (ICP < 20 mmHg and ICP ≥ 20 mmHg). Panel (**B**) illustrated the correlation between ONSD and ICP measurements. Panel (**C**) presented the receiver operating characteristic curve for ONSD as a diagnostic test for increased ICP ≥ 20 mmHg, with an Area Under the Curve (AUC) and the optimal threshold marked in red determined by using the Youden Index.

The relationship between ONSD and invasive ICP measurements is illustrated in Figure 4. Patients with ICP ≥ 20 mmHg had significantly larger ONSD values than those with ICP < 20 mmHg (*p* = 2.8 × 10^−16^; Figure 4A). Spearman’s correlation analysis demonstrated a moderate positive correlation between ONSD and invasive ICP across all measurement time points (rho = 0.49; 95% CI: 0.39–0.57; *p* < 2.2 × 10^−16^; Figure 4B). This correlation was particularly evident in measurements where ICP ≥ 20 mmHg (highlighted in red in Figure 4B), confirming that higher ONSD values consistently corresponded with elevations in invasive ICP.

The ROC analyses were conducted at the measurement level using 274 paired ONSD–PI–ICP observations. Receiver operating characteristic (ROC) curve analysis demonstrated good diagnostic accuracy for ONSD in identifying ICP ≥ 20 mmHg (AUC = 0.83; Figure 4C). Because the ROC analysis was performed at the measurement level, diagnostic performance may be optimistic compared with patient-level validation. Using the Youden Index, the optimal ONSD threshold was 5.88 mm, which yielded a sensitivity of 81% and a specificity of 82%. These findings suggest that ONSD ultrasonography may be a useful non-invasive screening tool for detecting intracranial hypertension. However, because the ROC analysis was performed at the measurement level, the proposed threshold should be considered preliminary and requires patient-level and external validation.

### 3.4. Diagnostic and Prognostic Performance of TCD-Derived PI

**Figure 5:**
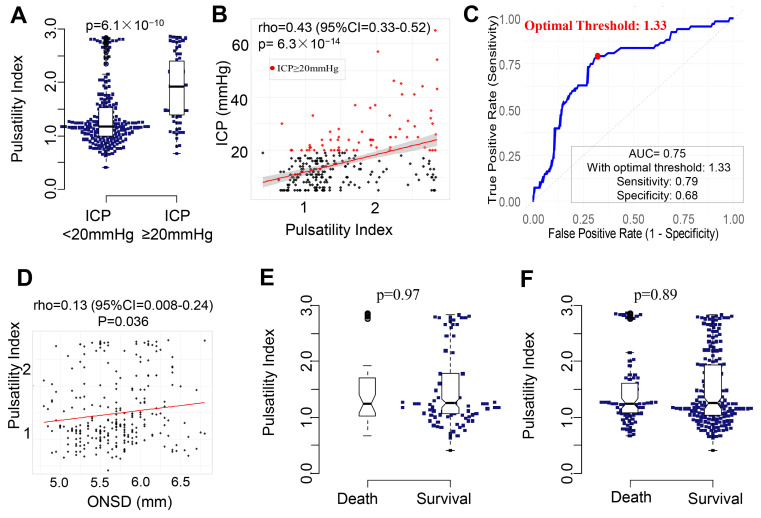
Diagnostic and prognostic value of pulsatility index (PI) in detecting elevated intracranial pressure (ICP) and mortality prediction. (**A**) Comparison of PI between patients with ICP < 20 mmHg and ICP ≥ 20 mmHg. (**B**) Scatter plot showing the correlation between PI and ICP, with red dots indicating ICP ≥ 20 mmHg. (**C**) receiver operating characteristic curve for PI in detecting ICP ≥ 20 mmHg, with an AUC of 0.75 and an optimal threshold of 1.33. (**D**) Scatter plot showing the correlation between PI and ONSD. (**E**,**F**) Box plots comparing PI values between survivors and non-survivors at 10 days (**E**) and 1 month (**F**) post-admission.

The diagnostic and prognostic utility of TCD-derived PI is summarized in Figure 5. Patients with ICP ≥ 20 mmHg demonstrated significantly higher PI values compared to those with ICP < 20 mmHg (*p* = 6.1 × 10^−10^; Figure 5A), confirming that PI elevation accompanies intracranial hypertension. Spearman’s correlation analysis revealed a moderate positive correlation between PI and invasive ICP (rho = 0.43; 95% CI: 0.33–0.52; *p* = 6.3 × 10^−14^; Figure 5B). ROC curve analysis demonstrated that PI achieved an AUC of 0.75 for detecting ICP ≥ 20 mmHg, with an optimal threshold of 1.33 (sensitivity: 79%; specificity: 68%; Figure 5C)—indicating moderate diagnostic accuracy, inferior to that of ONSD.

A weak but statistically significant correlation was identified between PI and ONSD (rho = 0.13; 95% CI: 0.008–0.24; *p* = 0.036; Figure 5D), suggesting that, while the two modalities capture partially overlapping aspects of ICP physiology, they remain largely complementary rather than interchangeable. Regarding prognostic utility, no significant difference in PI values was observed between patients who died within 10 days and those who survived (*p* = 0.97; Figure 5E), nor between patients who died within 30 days and survivors (*p* = 0.89; Figure 5F). These results indicate that PI alone lacks sufficient discriminatory power for early mortality prediction in this cohort.

### 3.5. Integrated Prediction of Elevated ICP and Early Mortality

**Figure 6:**
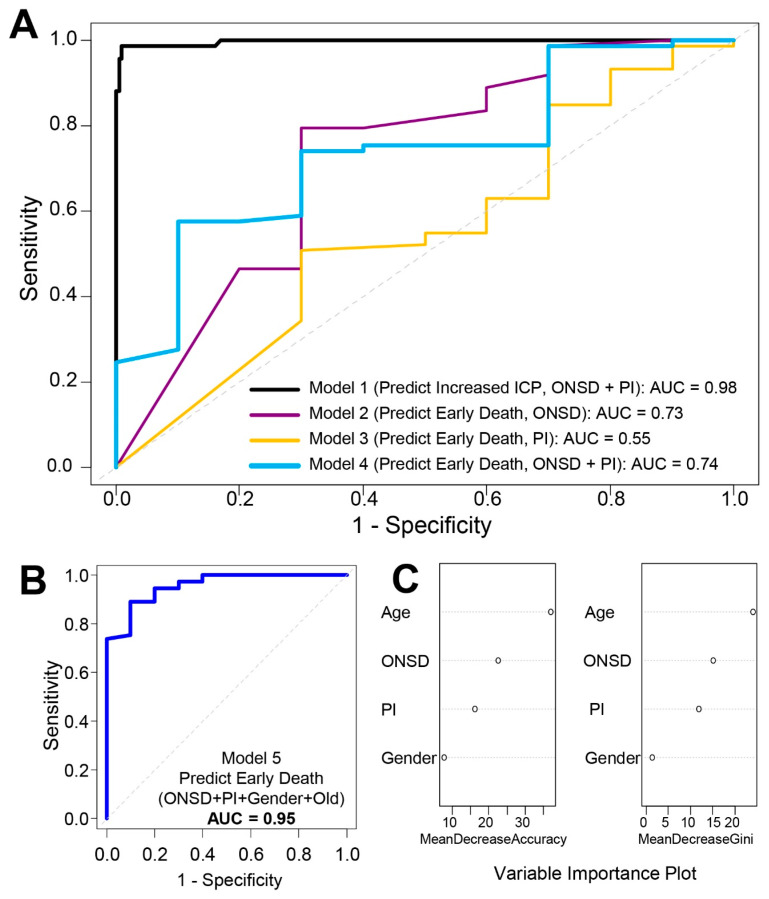
Prognostic models for increased ICP and early mortality. (**A**) ROC curves for different models predicting increased ICP and early mortality. Model 1 (black): ICP prediction with factors ONSD and PI. Model 2 (purple): Early mortality prediction using ONSD alone. Model 3 (yellow): Early mortality prediction using PI alone. Model 4 (blue): Early mortality prediction using the combination of ONSD and PI. (**B**) ROC curve for Model 5 to predict early mortality, integrating simple factors: ONSD, PI, age, and gender. (**C**) Variable importance plots for Model 5, showing Mean Decrease Accuracy (left) and Mean Decrease Gini (right).

To explore the additive value of combining ONSD and PI with simple clinical variables, five random forest models were evaluated (Figure 6). The ONSD and PI model showed high apparent discrimination for elevated ICP detection (AUC = 0.98). For early mortality prediction, ONSD alone showed moderate apparent discrimination (AUC = 0.73), whereas PI alone showed poor discrimination (AUC = 0.55). Adding PI to ONSD resulted in only modest improvement (AUC = 0.74). The model incorporating ONSD, PI, age, and sex showed the highest apparent discrimination for early mortality prediction (AUC = 0.95), with age and ONSD identified as the most influential predictors.

However, these machine learning results should be interpreted cautiously because the analysis was exploratory, the sample size was limited, repeated measurements were analyzed at the measurement level, and only nine early deaths occurred. Therefore, the reported model performance should be considered hypothesis-generating rather than definitive evidence of clinical predictive utility.

## 4. Discussion

This prospective study suggests that ONSD ultrasonography and TCD-derived PI may serve as complementary non-invasive markers of elevated ICP in patients with severe acute ICH. ONSD showed better diagnostic discrimination than PI, whereas PI provided additional hemodynamic information. However, because the study included only 42 patients and repeated measurements were analyzed at the measurement level, the findings should be interpreted as exploratory and require validation in larger independent cohorts.

### 4.1. ONSD Variability in Acute ICH

Prior studies have reported variations in ONSD values across different healthy populations. For instance, Maude et al. found a median ONSD of 4.41 mm in healthy Bangladeshis [16], while Bauerle et al. reported the mean ONSD was 5.4 ± 0.6 mm with a range of 4.3–7.6 mm in Germans [17]. In Asian populations, Wang et al. noted an ONSD range of 2.65–4.30 mm among 230 healthy Chinese adults [18], whereas Kim et al. documented a mean ONSD of 4.71 mm in healthy South Koreans [19]. A meta-analysis by Ertl et al. suggested that normal ONSD values range from 4.9 mm to 5.3 mm [20]. The ethnic variability in ONSD measurements underscores the necessity of region-specific reference values, such as those for Vietnamese populations. Our study focused exclusively on patients with ICH. The mean ONSD value obtained from 274 measurements across 42 patients was 5.70 mm (range: 4.80–6.80 mm), which is notably higher than reported normal values in healthy populations across studies. This difference underscores the potential impact of intracranial pathology on ONSD measurements and reinforces its role as a marker of elevated intracranial pressure in critically ill patients.

The absence of a significant correlation between ONSD at T_0_ and admission clinical severity scores, including GCS, NIHSS, hematoma volume, and Graeb score—warrants specific discussion, as this finding is counterintuitive but mechanistically coherent. Clinical severity scales in ICH primarily reflect the anatomical distribution and volume of hemorrhage, as well as the degree of brainstem compression, whereas ONSD reflects the net hydrostatic pressure transmitted to the optic nerve sheath via the subarachnoid space. These are distinct pathophysiological dimensions: a patient may present with a high Graeb score and severe neurological impairment yet maintain relatively normal ICP transiently if compensatory mechanisms (e.g., CSF redistribution, cerebral venous compliance) remain intact at the moment of admission. Conversely, an initially smaller hemorrhage in eloquent periventricular locations may generate disproportionate ICP elevation. This dissociation suggests that ONSD should not be regarded as a redundant correlate of clinical scoring, but rather as an independent, complementary physiological parameter that adds ICP-specific information beyond what conventional bedside assessments provide.

### 4.2. ONSD as a Non-Invasive Surrogate Marker of ICP

In this study, ONSD correlated moderately with ICP (rho = 0.49, *p* < 0.0001), consistent with previous findings but lower than some reports. For example, Moretti et al. (2009) found a stronger correlation (r = 0.7, 95% CI: 0.585–0.7936) in ICH patients [21], and Geeraerts et al. reported an r-value of 0.71 (*p* < 0.0001) in severe traumatic brain injury (TBI) cases [22]. Conversely, Frumin et al. observed a moderate correlation (r = 0.408, *p* = 0.03) in patients with external ventricular drainage [23]. ROC curve analysis in the present cohort revealed that ONSD (AUC = 0.83) demonstrated superior predictive accuracy for ICP elevation compared to PI (AUC = 0.75), consistent with prior studies [24]. This finding aligns with a previous study demonstrating that ONSD had the highest ability to detect intracranial hypertension, with an area under the curve (AUC) of 0.91 (95% CI: 0.88–0.95) [25]. The optimal ONSD threshold of 5.88 mm (sensitivity 81%, specificity 82%) derived in the present study is consistent with the empirically derived range of 5.7–6.0 mm reported across heterogeneous critical care populations, lending external validity to this cut-off despite the single-center design [24,26].

The convergence of the present threshold (5.88 mm) with the 5.7–6.0 mm range reported in predominantly European and mixed-ethnicity critical care cohorts is notable, given that baseline normative ONSD values in Asian populations are systematically lower than in European populations. This convergence implies that ICP-driven mechanical distension of the optic nerve sheath overrides ethnic baseline differences once pathological ICP levels are sustained—a phenomenon consistent with the non-linear compliance characteristics of the sheath. At low-to-normal ICP, sheath compliance buffers pressure transmission, resulting in minimal ONSD change; beyond a critical ICP threshold (approximately 15–20 mmHg), compliance is exhausted and ONSD increases steeply. This threshold effect may explain why diagnostic cut-offs cluster within a narrow range (5.5–6.0 mm) regardless of population, and it supports the provisional applicability of the 5.88 mm threshold in Vietnamese ICH patients pending population-specific validation. The ONSD threshold of 5.88 mm identified in this study should be interpreted as a preliminary, cohort-derived cut-off. Although it showed clinically meaningful diagnostic performance in this prospective cohort, external validation in larger multicenter populations is required before it can be recommended as a definitive clinical decision threshold.

### 4.3. Diagnostic Role of TCD-Derived PI

Spearman’s correlation analysis identified a moderate positive correlation between PI and invasive ICP (rho = 0.43; *p* < 0.001). Our study confirmed its diagnostic utility but reaffirmed that it is a less reliable predictor of ICP compared to ONSD [9]. Zweifel et al. reported a weaker correlation (r = 0.31, *p* < 0.001) [7], while Robba et al. found PI to be an unreliable continuous ICP monitoring tool [6]. However, Moreno et al. reported a strong correlation (PI = 0.48 + 0.03 × ICP, r = 0.69, *p* < 0.0001). The variability in PI and ICP correlations may stem from differences in patient populations, ICP measurement techniques, and hemodynamic factors. One main reason for this variability is the location of ICP measurement. Ventricular ICP, considered the gold standard for global ICP assessment, may differ from parenchymal ICP, which reflects more localized pressure changes [27,28]. Since most of our cohort (88.1%) had ventricular ICP monitoring, our findings likely reflect a more global representation of ICP rather than focal changes. Unlike ONSD, which directly reflects cerebrospinal fluid pressure, PI represents cerebrovascular resistance and is influenced by systemic hemodynamics, making it a less direct but still valuable indicator of ICP.

The exclusion of patients without adequate temporal bone acoustic windows may limit the generalizability of PI-based assessment. Because TCD feasibility depends on anatomical and skull-related factors, PI monitoring may not be applicable to all patients with severe ICH. Future studies should compare included and excluded patients and evaluate alternative insonation strategies or complementary non-invasive modalities.

Of particular note, the absence of a significant difference in PI values between patients who died within 10 days and those who survived (*p* = 0.97) represents a meaningful negative finding. PI, as a single time-point waveform index, reflects instantaneous cerebrovascular resistance rather than the cumulative burden of secondary injury that ultimately determines survival. Mortality in severe ICH is determined by the interaction of hematoma expansion, IVH progression, cerebral oedema, and systemic complications over days—dynamics that are not captured by discrete PI measurements. This diagnostic-prognostic dissociation argues against using PI in isolation as a mortality risk stratification tool, while affirming its value as a real-time ICP surrogate when used in conjunction with ONSD.

### 4.4. Complementary Value of ONSD and PI

ONSD directly reflects CSF pressure transmitted to the optic nerve sheath, serving as a static marker of ICP elevation. In contrast, TCD-derived PI evaluates dynamic cerebral hemodynamic changes in response to rising ICP. Standalone measurements have inherent limitations. ONSD is subject to inter-operator variability and baseline anatomical differences, while PI may be affected by extracranial arterial disease and insonation difficulties. Combining these modalities mitigates these issues, enhancing diagnostic accuracy. ONSD and TCD-derived PI should therefore be interpreted as complementary rather than interchangeable markers of intracranial pathophysiology. Our study showed that using both ONSD and PI improved agreement with invasive ICP monitoring and was consistent with findings from the SYNAPSE-ICU study, which demonstrated enhanced ICP estimation accuracy with multimodal approaches [29]. Robba et al. also found that the combination of ONSD and venous transcranial Doppler (vTCD) of straight sinus systolic flow velocity showed a statistically significant improvement of AUC values compared with the ONSD method alone (0.93, 95% CI 0.90–0.97, *p* = 0.01) [2].

The weak but statistically significant inter-modality correlation (rho = 0.13; *p* = 0.036) between ONSD and PI deserves careful interpretation. This low correlation does not indicate poor measurement quality; rather, it quantifies the degree to which the two modalities capture distinct physiological processes. ONSD reflects a quasi-static, anatomically mediated pressure signal integrated over the CSF compartment, while PI captures moment-to-moment pulsatile haemodynamic changes modulated by cerebrovascular autoregulation. The low shared variance (~1.7%) between the two indices confirms that they are genuinely complementary rather than redundant. From a clinical decision-making standpoint, this means that a patient with normal ONSD but elevated PI (reflecting early autoregulatory impairment before sheath distension occurs) would be identified by PI but not ONSD, and vice versa. The combination thus expands the diagnostic window to capture both static pressure overload and dynamic vascular dysregulation—two distinct mechanisms of secondary brain injury in ICH.

### 4.5. Integrated Prediction of Early Mortality

The exploratory machine learning analysis suggested that combining ultrasound-derived markers with simple demographic variables may improve early mortality risk stratification. In particular, age and ONSD appeared to be the most influential predictors in the model incorporating ONSD, PI, age, and sex. This is biologically plausible, as age reflects baseline physiological reserve, while ONSD reflects the burden of intracranial hypertension. The demographic imbalance of the cohort may limit the generalizability of the findings. Most patients were aged 50–69 years and were male, which may reduce the ability to assess age- or sex-specific effects.

Nevertheless, these findings should be interpreted with substantial caution. Only nine patients died within 10 days, and the model was developed using a small repeated-measurement dataset. In addition, no external validation, leave-one-subject-out cross-validation, class-weighted random forest, or synthetic oversampling approach was performed. Therefore, the machine-learning results should be considered exploratory and hypothesis-generating rather than clinically actionable.

Nevertheless, these findings should be interpreted with substantial caution. Only nine patients died within 10 days, and the model was developed using a small repeated-measurement dataset. In addition, no external validation, leave-one-subject-out cross-validation, class-weighted random forest, or synthetic oversampling approach was performed. Therefore, the machine learning results should be considered exploratory and hypothesis-generating rather than clinically actionable, and leave-one-subject-out cross-validation or external validation is required to assess generalizability to unseen patients.

Machine learning models demonstrated that combining ONSD and PI may improve early mortality risk stratification, although this finding remains exploratory and vulnerable to overfitting because of the small number of early mortality events. Specifically, Model 4 (ONSD + PI; early mortality prediction) achieved an AUC of 0.74, while Model 5, incorporating age and sex, further enhanced predictive accuracy (AUC = 0.95), highlighting the importance of a multimodal approach. Among the predictors, ONSD emerged as the most influential factor, reinforcing its role as a key marker of mortality risk. These findings align with Sykora et al. (2014), who identified elevated PI as an independent predictor of poor functional outcomes in ICH [9].

Variable-importance analysis identified age and ONSD as the dominant predictors, whereas PI contributed modestly and sex had minimal independent influence. The strong contribution of age is biologically plausible and consistent with established ICH prognostic frameworks, as age may reflect baseline cerebrovascular reserve, frailty, and reduced capacity for neurological recovery beyond the acute ICP burden. In contrast, the limited contribution of sex should be interpreted cautiously, given the marked male predominance in this cohort, which reduces the ability to detect sex-specific effects. Although the high AUC of Model 5 suggests that combining physiological biomarkers with simple clinical variables may improve early mortality stratification beyond biomarker-only models, these findings remain exploratory. The small number of early deaths within 10 days limits model stability, and external validation in larger independent cohorts is required before clinical implementation.

### 4.6. Clinical Implications and Future Directions

The 2022 AHA/ASA ICH guidelines acknowledge that while invasive ICP monitoring provides precise, continuous data to guide osmotherapy and surgical interventions, its benefit-risk profile in spontaneous ICH—as opposed to TBI—remains incompletely defined [1]. A retrospective analysis by Menacho et al. (2021) reported that ICP monitor-guided therapy was independently associated with worse neurological outcomes in spontaneous non-traumatic ICH (OR = 2.76, *p* = 0.008), likely reflecting the confounding effect of clinical severity on the decision to place a monitor, rather than a causal harm of monitoring per se [4]. Nevertheless, these data collectively motivate the development of non-invasive strategies that can guide ICP management without the risks associated with catheter placement.

The present findings suggest that serial ONSD measurement, supplemented by TCD-derived PI at selected clinical time points, may provide a feasible bedside strategy for non-invasive ICP surveillance in severe acute ICH, particularly in resource-limited neurocritical care settings where invasive ICP monitoring may be constrained by technical expertise, procedural risk, and equipment availability. This approach relies on portable ultrasound equipment and may serve as a practical complement, rather than a replacement, to invasive ICP monitoring.

The ONSD threshold of 5.88 mm and PI threshold of 1.33 identified in this study should be regarded as preliminary, hypothesis-generating cut-off values. Although they may help inform bedside risk assessment in similar clinical contexts, they should not be considered definitive clinical decision thresholds until validated in larger external cohorts.

Future research should prioritize three directions. First, prospective multi-center studies in Southeast Asian ICH populations are needed to validate ethnicity-specific ONSD thresholds and to establish region-specific normative baselines. Second, the integration of serial ONSD and PI trajectories (ΔT_0_→T_9_) into dynamic machine learning models may capture ICP evolution patterns predictive of outcome beyond single time-point values. Third, the combination of ONSD and PI with emerging non-invasive modalities—including automated infrared pupillometry (Neurological Pupil Index, NPI), near-infrared spectroscopy (NIRS), and non-invasive ICP estimation from TCD waveform morphology—represents a promising multimodal strategy that warrants systematic evaluation in prospective adaptive trial designs.

### 4.7. Limitations

There were limitations that should be considered. First, this was a single-center study with a relatively small cohort of patients with severe acute ICH; therefore, the proposed ONSD and PI thresholds and machine learning models require external validation in larger multicenter cohorts. Second, although 274 paired ONSD–PI–ICP measurements were analyzed, they were derived from only 42 patients and were therefore not statistically independent. This repeated-measurement structure may have introduced within-subject correlation and affected correlation estimates, *p*-values, ROC performance, and machine-learning metrics. Future studies should use mixed-effects models, generalized estimating equations, or other clustered approaches to account for intra-subject dependence. Third, the ROC analyses were performed at the measurement level; therefore, diagnostic performance may have been overestimated compared with patient-level validation. Fourth, the machine learning analyses were exploratory and vulnerable to overfitting because of the small sample size, limited number of early deaths, and potential class imbalance. No external validation, leave-one-subject-out cross-validation, class-weighted random forest, or synthetic oversampling approach was performed. Future studies should use subject-wise validation and class-sensitive metrics such as precision, recall, F1-score, and precision–recall curves to better assess detection of clinically dangerous ICP elevation. Fifth, formal inter- and intra-observer reliability analyses were not performed. Although all ultrasound measurements followed a standardized protocol and were performed by trained clinicians, operator-dependent variability cannot be excluded. Sixth, most ICP measurements were obtained from ventricular catheters, while only a small proportion were obtained from intraparenchymal probes; therefore, the influence of catheter location on the ultrasound–ICP relationship could not be fully assessed. Finally, the study focused on early mortality, while longer-term functional outcomes and dynamic trajectories of ONSD and PI require further investigation.

## 5. Conclusions

The present prospective pilot study suggests that ONSD ultrasonography and TCD-derived PI may serve as complementary non-invasive markers for ICP assessment in patients with severe acute ICH. ONSD demonstrated superior diagnostic accuracy for detecting elevated ICP (AUC = 0.83; optimal threshold: 5.88 mm), whereas PI provided additional hemodynamic information. Their combination in an exploratory random forest model showed high apparent discrimination for elevated ICP detection (AUC = 0.98). In addition, integrating these physiological biomarkers with age and sex appeared to improve early mortality prediction (AUC = 0.95), with ONSD emerging as the dominant predictors. These findings suggest that serial bedside ONSD and PI measurements may complement clinical assessment and invasive ICP monitoring when available. However, because of the small cohort size, repeated-measurement structure, limited number of early deaths, and absence of external validation, the proposed thresholds and machine learning models should be considered preliminary. Larger independent cohorts with patient-level validation are required before these findings can be recommended for routine clinical decision-making.

## Figures and Tables

**Table 1:** Demographic, clinical, and radiological characteristics of 42 patients with acute intracerebral hemorrhage.

Characteristics	n (%)	Characteristics	n (%)
**Demographic features**		**Hemorrhage categories**	
**age group (years)**		Parenchymal hemorrhage	27 (64.3)
<50	8 (19)	Intraventricular hemorrhage	40 (95.2)
50–69	26 (61.9)	Subarachnoid hemorrhage	14 (33.3)
>70	8 (19)	Fisher Grade 4	14 (100)
**Gender**		**Graeb Score**	
Male	36 (85.7)	Mild IVH (Graeb Score ≤ 4)	9 (21.4)
Female	6 (14.3)	Moderate IVH (Graeb Score 5–7)	16 (38.1)
**Comorbidities**		Severe IVH (Graeb Score ≥ 8)	17 (40.5)
Hypertension	36 (85.7)	**Cerebrovascular abnormalities**	
Diabetes mellitus	11 (26.2)	Cerebral aneurysm	11 (26.2)
Kidney Failure	2 (4.8)	Arteriovenous malformation (AVM)	1 (2.4)
Liver cirrhosis	2 (4.8)	Moyamoya disease	1 (2.4)
History of stroke	5 (11.9)	No detection of abnormalites	29 (69.0)
**Glasgow score at admission**		**Surgical and Interventional Treatments**	
GCS: 3–8 (Severe)	30 (71.4)	Surgical external ventricular drainage (EVD)	36 (85.7)
GCS: 9–12 (Moderate)	11 (28.6)	Hematoma drainage (HD)	4 (9.5)
**NIHSS score at admission**		Combined HD and EVD	1 (2.4)
NIHSS: ≤4 (minor stroke)	2 (4.8)	Endovascular treatment	1 (2.4)
NIHSS: 5–15 (Moderate stroke)	7 (16.6)	**Probe placement sites for ICP monitoring**	
NIHSS: 16–20 (Moderate to severe stroke)	5 (11.9)	Ventricle	37 (88.1)
NIHSS: 21–42 (Severe stroke)	28 (66.7)	parenchyma	5 (11.9)
**Modified Rankin Scale (mRS)**		**Clinical outcome**	
mRS: 1–3 (Independent or minimal disability)	2 (4.8)	Early death within 10 days from onset	9 (21.4)
mRS: 3–5 (Significant disability, assistance)	25 (59.5)	Early death within 30 days from onset	15 (35.7)
mRS = 6 (Mortality)	15 (35.7)	Survival after 30 days from onset	27 (64.3)

Abbreviations: GCS, Glasgow Coma Scale; NIHSS, National Institutes of Health Stroke Scale; IVH, intraventricular hemorrhage; SAH, subarachnoid hemorrhage; AVM, arteriovenous malformation; EVD, external ventricular drainage; HD, hematoma drainage; ICP, intracranial pressure; mRS, modified Rankin Scale. Data are presented as number and percentage [n (%)].

## Data Availability

The data generated and analyzed during the current study are available from the corresponding author upon reasonable request. The data are not publicly available due to privacy and ethical restrictions related to patient-level clinical and monitoring data.

## Abbreviations

The following abbreviations are used in this manuscript:

AVM	Arteriovenous malformation
EVD	External ventricular drainage
GCS	Glasgow score
HD	Hematoma drainage
ICH	Intracerebral hemorrhage
ICP	Intracranial pressure
MCA	Middle cerebral artery
mRS	Modified Rankin scale
NIHSS	NIH stroke scale
ONSD	Optic nerve sheath diameter
PI	Pulsatility index
SAH	Subarachnoid hemorrhage
TCD	Transcranial Doppler
